# Regulatory challenges with biosimilars: an update from 20 countries

**DOI:** 10.1111/nyas.14522

**Published:** 2020-11-21

**Authors:** Hye‐Na Kang, Robin Thorpe, Ivana Knezevic, Mary Casas Levano, Mumbi Bernice Chilufya, Parichard Chirachanakul, Hui Ming Chua, Dina Dalili, Freddie Foo, Kai Gao, Suna Habahbeh, Hugo Hamel, Gi Hyun Kim, Violeta Perez Rodriguez, Desi Eka Putri, Jacqueline Rodgers, Maria Savkina, Oleh Semeniuk, Shraddha Srivastava, João Tavares Neto, Meenu Wadhwa, Teruhide Yamaguchi

**Affiliations:** ^1^ Department of Health Product Policy and Standards World Health Organization Geneva Switzerland; ^2^ Independent Expert Welwyn United Kingdom; ^3^ General Directorate of Medicines Supplies and Drugs (DIGEMID) San Miguel Peru; ^4^ Zambia Medicines Regulatory Authority Lusaka Zambia; ^5^ Food and Drug Administration Nonthaburi Thailand; ^6^ National Pharmaceutical Regulatory Agency Selangor Malaysia; ^7^ Iran Food and Drug Administration Tehran Iran; ^8^ Health Sciences Authority Singapore; ^9^ Shanghai University Shanghai People's Republic of China; ^10^ Jordan Food and Drug Administration Amman Jordan; ^11^ Health Canada Ottawa Ontario Canada; ^12^ Ministry of Food and Drug Safety Osong‐eup Republic of Korea; ^13^ Centro para el Control Estatal de Medicamentos Equipos y Dispositivos Médicos (CECMED) La Habana Cuba; ^14^ The Indonesian Food and Drug Authority Jakarta Indonesia; ^15^ Food and Drugs Authority Accra Ghana; ^16^ The Federal State Budgetary Institution “Scientific Centre for Expert Evaluation of Medicinal Products” of the Ministry of Health Moscow Russian Federation; ^17^ Ministry of Health of Ukraine Kyiv Ukraine; ^18^ Central Drug Standards Control Organization (CDSCO) Ministry of Health & Family Welfare New Delhi India; ^19^ Brazilian Health Regulatory Agency (ANVISA) Brasilia Brazil; ^20^ National Institute for Biological Standards and Control, Medicines and Healthcare products Regulatory Agency Potters Bar United Kingdom; ^21^ Pharmaceuticals and Medical Devices Agency Tokyo Japan

**Keywords:** biosimilar, regulation, challenges, survey, WHO

## Abstract

The World Health Organization (WHO) issued guidelines for the regulatory evaluation of biosimilars in 2009 and has provided considerable effort toward helping member states implement the evaluation principles in the guidelines into their regulatory practices. Despite this effort, a recent WHO survey (conducted in 2019–2020) has revealed four main remaining challenges: unavailable/insufficient reference products in the country; lack of resources; problems with the quality of some biosimilars (and even more with noninnovator products); and difficulties with the practice of interchangeability and naming of biosimilars. The following have been identified as opportunities/solutions for regulatory authorities to deal with the existing challenges: (1) exchange of information on products with other regulatory authorities and accepting foreign licensed and sourced reference products, hence avoiding conducting unnecessary (duplicate) bridging studies; (2) use of a “reliance” concept and/or joint review for the assessment and approval of biosimilars; (3) review and reassessment of the products already approved before the establishment of a regulatory framework for biosimilar approval; and (4) setting appropriate regulatory oversight for good pharmacovigilance, which is essential for the identification of problems with products and establishing the safety and efficacy of interchangeability of biosimilars.

## Introduction

One of the core functions of the World Health Organization (WHO) is to set norms and standards and promote and monitor their implementation. A range of standards (e.g., recommendations and guidelines) that support regulation of biologicals worldwide have been developed by the WHO to assist its 194 member states. The WHO guidelines on the evaluation of similar biotherapeutic products (SBPs; hereafter referred to as the Guidelines)^1^ were adopted by the WHO Expert Committee on Biological Standardization in 2009. Since then, the WHO has provided considerable effort toward helping member states implement the evaluation principles in the Guidelines into their regulatory practices.^2^ Despite this effort, several regulatory challenges still remain.^3^, ^4^, ^5^ A survey conducted previously in 2010 revealed three main challenges, namely: (1) appropriate comparability studies had not been recommended by regulators in some countries; (2) some products had been inappropriately called *biosimilars* without conducting full comparability studies for their approval; and (3) the term *biogeneric* products was prevalent and in use along with inappropriate use of the term *biosimilar* in some countries.^6^ While some of these challenges have been resolved,^2^ ongoing or new challenges have arisen as an increasing number of biosimilars are evaluated and placed on the market.

This article describes the regulatory challenges identified by the survey participants from 20 countries in a recent WHO survey conducted in 2019 (and updated in 2020) and suggests potential solutions to overcome these challenges. This unique, comprehensive survey is detailed, covers most current topics pertinent to biosimilars, and allows an assessment on a global scale of the current situation with biosimilars and related issues.

## Methodology

The questionnaire for the survey prepared by the WHO was similar to that used for the previous (August 2010) survey^6^ but updated to include additional data, such as existence of noninnovator or me‐too products and the plan for reassessment. Regulatory experts in 20 countries were invited to participate in the survey. The countries included those that participated in the survey in 2010^6^ or were involved in the WHO implementation activities during the past 10 years.^2^ The assessment results presented here are based on the answers provided by survey participants from 18 countries in May–June 2019 and, in addition, from Singapore and China in November and December 2019, respectively. All responses were updated and confirmed in June 2020. The feedback from the UK refers to the situation in the EU rather than specifically for the United Kingdom.

## Results

### Originator and reference products

#### Originator products

The WHO guidelines define an originator product as a medicine that has been licensed by the national regulatory authorities (NRAs) on the basis of a full registration dossier, that is, the approved indication(s) for use were granted on the basis of full quality, efficacy, and safety data.^1^

Most countries (i.e., Brazil, Canada, China, Egypt, Ghana, India, Indonesia, Japan, Jordan, Malaysia, Republic of Korea, Russia, Singapore, and Thailand) and the EU accept the above definition for an originator product as they responded positively to the survey phrasing for this, that is, a product licensed by the local regulatory authority on the basis of submitted quality, nonclinical, and clinical data, gained through its own laboratory studies and clinical trials (full/complete data package). However, there were some slight differences in the wording preferred by some countries, for example Indonesia, but this did not significantly affect the meaning of the definition. Peru did not have a definition for an originator product in its regulation.

In addition, some countries approved originator products on the basis of the review of the full data package conducted by one or more of the well‐experienced regulatory authorities. In some countries, approval by another NRA serves as a reliable approach, while other countries regard it as additional available information when making their own assessment. In any case, each NRA has responsibility for its own decision regarding the approval of a product (see examples in Table 1).

**Table 1:** Acceptable reference countries/areas for originator products for countries that allow this use

Countries	Acceptable reference countries/areas
Cuba	Regional reference countries in the performance of the health regulatory functions recommended by PAHO, for example, Argentina, Brazil, Canada, Chile, Colombia, Cuba, Mexico, and the United States
India	Australia, Canada, the EU, Japan, the United States, and the WHO
Iran	EU and the United States
Jordan	Australia, Canada, EU (centralized procedure), Japan, and the United States
Ukraine	EU
Zambia	EU, Japan, Republic of Korea, the United States, and the WHO

The evidence from the survey showed that some countries have a list of reference countries from which they accept originator products that have been approved (see Table 1). The regulatory authorities have also used this list as recognized regulatory authorities as a basis for considering products licensed by these authorities as potential candidates for reference products (see Table 2).

**Table 2:** Acceptable RBPs: foreign‐licensed and ‐sourced RBPs

Countries	Criteria or accepted reference countries
Brazil	In case of proven commercial unavailability of RBP licensed and marketed locally, if having the similar technical scientific criteria to Agência Nacional de Vigilância Sanitária (ANVISA)'s criteria; and having possibility of full and unrestricted access to the registration information for ANVISA.
Canada	Sponsors may use a non‐Canadian sourced version as a proxy for the Canadian drug in the comparative studies. The onus is on the sponsor to demonstrate that the chosen reference biologic drug is suitable to support the submission. The non‐Canadian reference biologic drug should be marketed in a jurisdiction that formally adopts International Council for Harmonization (ICH) guidelines and that has regulatory standards and principles for evaluation of medicines, postmarket surveillance activities, and approaches to comparability that are similar to Canada.
Cuba	Countries or regions with experience in manufacturing control, regulation, and PMS of biological/biotechnological products. NRA declared as reference in the region of the Americas, according to the evaluation process established by the PAHO.
Egypt	Australia, Austria, Belgium, Canada, Denmark, Germany, Finland, France, Holland, Iceland, Ireland, Italy, Japan, Luxemburg, New Zealand, Norway, Portugal, Spain, Sweden, Switzerland, United States, and UK
Ghana	EU and the United States
India	ICH countries
Indonesia	Approved with established evaluation system and never rejected in Indonesia.
Iran	EU and the United States
Jordan	Australia, Canada, EU (via centralized procedure), Japan, and the United States
Malaysia	Canada, EU, France, Japan, Sweden, Switzerland, UK, and the United States
Peru	Australia, Austria, Belgium, Canada, EU (via centralized procedure), Denmark, France, Germany, Hungary, Ireland, Italy, Japan, Netherlands, Norway, Portugal, Republic of Korea, Spain, Sweden, Switzerland, the United States, and UK
Thailand	With the permission stated in a Thai FDA announcement.
Ukraine	EU (via centralized procedure) and the United States
Zambia	EU, Japan, Republic of Korea, United States, and the WHO

ANVISA, Agência Nacional de Vigilância Sanitária; PAHO, Pan American Health Organization.

#### Reference biotherapeutic products and their choice

A reference biotherapeutic product (RBP) is used as the comparator for head‐to‐head comparability studies with the SBP (also called biosimilar) in order to show similarity in terms of quality, safety, and efficacy.^1^ Only an originator product that was licensed on the basis of a full registration dossier can serve as an RBP. An SBP should, therefore, not be chosen as an RBP.^1^

The RBP is central to the licensing of an SBP, and the choice of a suitable RBP is fundamental for biosimilar development. The RBP should have been marketed for a suitable duration, have a significant volume of marketed use in the relevant country or area, and have a long established history of good safety and efficacy.^1^

#### Three scenarios in the acceptance/choice of RBP

*Use of domestically licensed and sourced RBP*. Regulatory authorities in all survey participating countries prefer that the comparability of the biosimilar and its RBP will be demonstrated using the domestically (nationally and locally) licensed and sourced product. This is because the regulatory authority in the jurisdiction concerned will already be familiar with this product and its clinical use.^7^

##### Use of foreign licensed and sourced RBP

In general, regulatory authorities may require the use of a domestically licensed RBP for licensing of the SBP. However, this practice may not be feasible for countries that lack particular nationally licensed originator products (e.g., Ghana, Peru, and Zambia) and regulatory authorities may need to consider establishing additional criteria to guide the acceptability of using an RBP licensed and sourced in other countries.^1^

The Guidelines anticipated this eventuality and proposed that in such cases the RBP should be licensed and widely marketed in another jurisdiction that has a well‐established regulatory framework and principles, as well as considerable experience with evaluation of biotherapeutic products and postmarketing surveillance activities.^1^ The survey identified that regulatory authorities of many countries (i.e., Cuba, Egypt, Ghana, India, Indonesia, Iran, Jordan, Malaysia, Peru, Thailand, Ukraine, and Zambia) have established a list of recognized regulatory authorities as a basis for considering products licensed by these authorities as potential candidates for RBPs (see Table 2). In Brazil, in cases of proven commercial unavailability of RBPs licensed and marketed locally, the product to be used in the comparability exercise must be previously discussed and agreed by the NRA. In this case, the RBP must be licensed by an authority that adopts technical and scientific criteria similar to those of the Brazilian authority and there must be the possibility of full and unrestricted access to original licensing information. The acceptance of an RBP for evaluation of an SBP in a particular country does not imply that the regulatory authority of that country has approved the RBP for use.^1^ The survey also identified RBPs that are not domestically approved (i.e., there is no licensed originator product) but have been used as the comparator for SBP approval in some countries. This process clearly requires the acceptability of foreign licensed and sourced RBPs, as is the case in several countries (see Table 2). This is important as it contributes to expanding the availability of various product classes since these products were not available on the market before approval of the SBP. Examples of this latter type of situation are:

- In Cuba, Omnitrope® (an SBP somatropin) was licensed in 2014, but its RBP (Genotropin®) has never been licensed in Cuba as an originator product.
- In Egypt, a similar situation arose as the above, with Omnitrope being approved in 2016.
- In Ghana, SBP epoietin alfas were approved (Binocrit® and REPOEITIN®) without the RBP EPREX® being licensed.
- In Indonesia, an SBP epoietin alfa was approved (Epoglobin®) in 2006 without the approval of the Epogen® RBP. The insulin SBP Insuman was licensed in 2012 although the RBP (Humulin) is not approved.
- In Peru and Zambia, a total of four and eight SBPs, respectively, were approved by August 2019 (see Ref. 2) for which respective RBPs were not licensed.

##### Use of domestically licensed but foreign sourced RBP or more than one sourced RBP

It is recommended that the same RBP should be used throughout the development of the SBP, that is, throughout the comparative quality, nonclinical, and clinical studies.^1^ Nevertheless, when the manufacturer plans the global development of an SBP and wishes to avoid unnecessary repetition of nonclinical and clinical studies already undertaken with an RBP licensed and sourced in another country, the use of a domestically licensed RBP for the quality studies and a foreign‐sourced RBP in nonclinical and clinical comparability studies may be possible, if justified.^7^ For example, the manufacturer may demonstrate that the domestically licensed RBP and the foreign‐sourced RBP are versions of the same RBP.^7^

The survey revealed that Russia does not accept the use of foreign‐sourced RBPs, but other countries accept their use, with some restrictions on their terms for acceptance. Cuba, Egypt, India, Iran, Jordan, Peru, Ukraine, and Zambia accept the use of foreign‐sourced RBPs based on publicly available technical, scientific, and clinical data with supporting evidence (i.e., paper‐based evidence) on comparability between local‐licensed and foreign‐sourced RBPs provided by the SBP manufacturer. Ghana and Singapore have adopted a flexible approach that requires bridging studies, unless this is considered scientifically unnecessary (i.e., if it can be justified by dossier assessment that the study is not required), but Brazil, Canada, China, the EU, Indonesia, Japan, Malaysia, Republic of Korea, and Thailand always require bridging studies for this. Table 3 shows the use of more than one RBP throughout comparability exercises to demonstrate the similarity of biosimilars by requiring bridging studies (such as analytical and even clinical pharmacokinetic/pharmacodynamic studies) between two RBPs (domestically licensed compared with foreign‐sourced RBP) or among three products (domestically licensed RBP compared with foreign‐sourced RBP and both compared with the biosimilar candidate).

**Table 3:** Acceptable RBPs: variously sourced RBPs

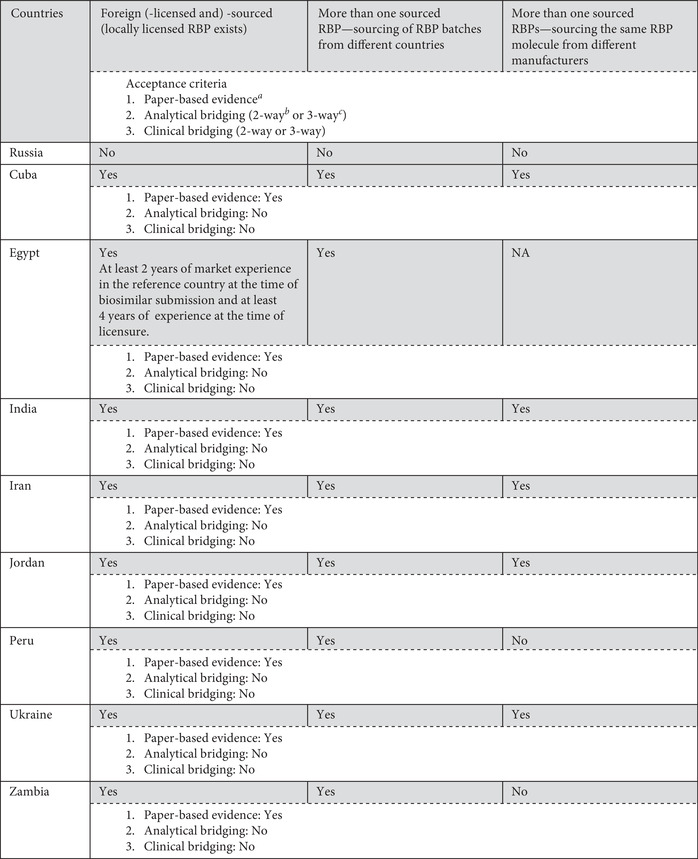
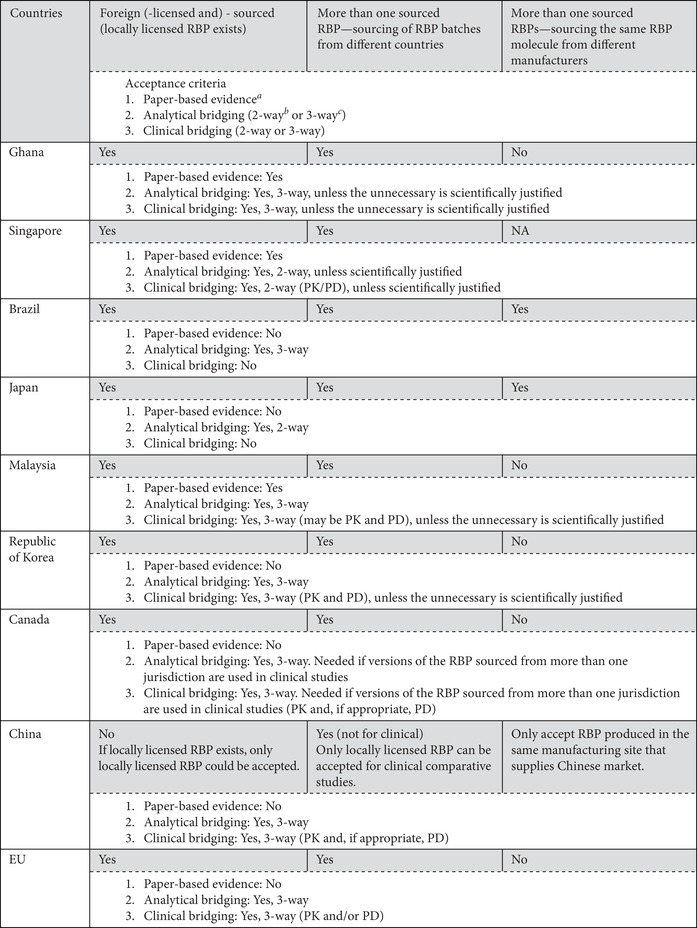
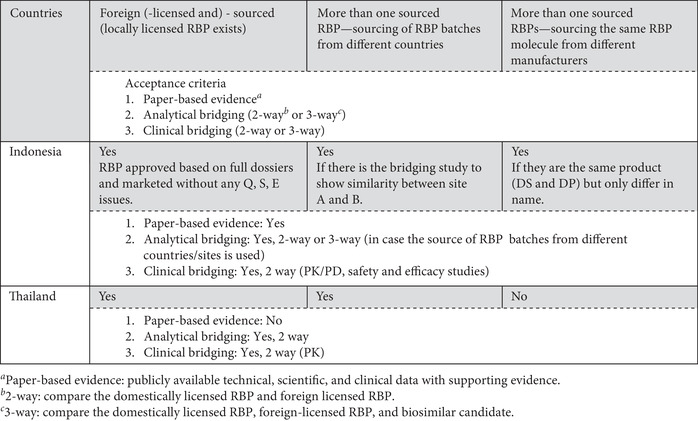

### Biosimilars and other noninnovator products

#### Approval of biosimilars

All survey‐participating countries now have biosimilar guidelines in place and biosimilars approved, although the date of adoption of guidance and numbers of biosimilars licensed varies considerably between countries/regions.^2^ In general, the decision to approve biosimilars is made based on the regulatory authority's own assessment of a full comparability exercise, including data from comprehensive quality, nonclinical, and clinical studies. One exception to this is in Cuba for monoclonal antibodies with cancer indication(s), where noncomparative clinical data may be considered, but on a case‐by‐case basis.

Some countries use a reliance concept and/or joint review for the decision to approve biosimilars. For the former, a regulatory authority recognizes or relies on the decisions of another regulatory authority based on previous expert review where the biosimilar is already licensed. This requires regulatory authorities adopting this approach to formulate a list of other regulatory authorities whose approvals they will recognize. In some cases, joint review with other regulatory authorities is sometimes undertaken. See Tables 4 and 5 for a listing of examples of such activities.

**Table 4:** Regulatory collaboration used for biosimilar approval

Countries	Reliance: countries where the NRA approval is to be recognized	Joint review
Canada	None	Feasibility is under discussion among the ACSS (Australia, Canada, Singapore, Switzerland) Consortium
Ghana	EU and the United States	Economic Community of West African States (ECOWAS)*a* WHO Collaborative Registration Procedure (CRP) and WHO Prequalification (PQ)
India*b*	Australia, Brazil, Canada, the EU, Japan, UK, and the United States	No
Iran	EU and the United States	No
Jordan*c*	Experienced regulatory authorities (e.g., the EU and the United States)	No
Malaysia	EU and the United States	No
Peru	Canada, the EU, ICH, PANDRH, the United States, and the WHO	No
Singapore	None	Feasibility is under discussion among the ACSS (Australia, Canada, Singapore, Switzerland) Consortium
Zambia	EU, the United States, and the WHO (SRA PQ procedure)	ZAZIBONA collaborative procedure*d* WHO PQ Article 58 of Regulation (EC)*e*

^*a*^ Member states of ECOWAS: Benin, Burkina Faso, Cabo Verde, Cote D'ivoire, the Gambia, Ghana, Guinea, Guinea Bissau, Liberia, Mali, Niger, Nigeria, Senegal, Sierra Leone, and Togo.

^*b*^ In case of orphan drug.

^*c*^ Fast‐track procedure.

^*d*^ NRAs from participating countries, namely, South Africa, Zimbabwe, Namibia, Botswana, and so on, have jointly reviewed dossiers with the Zambian NRA (Zambia Medicines Regulatory Authority).

^*e*^ For certain products, assessment by EMA would help if Zambia Agency is cohort in the reviews under article 58 procedure.

ICH, International Council for Harmonization; PANDRH, Pan American Network for Drug Regulatory Harmonization.

**Table 5:** Concept of reliance/recognition, that is, full reliance on the assessment of others versus additional assessment to be done by the local NRA

Countries	Full reliance on the assessment by other NRAs	Additional assessment by the local NRA
Ghana*a*	Yes	Stability data, particularly in‐use stability Verification of administrative information, for example, label, package insert, SmPC, RMP, and PhV plan
India	Yes	Phase IV study assessment
Iran	Yes	CTD review, laboratory testing, GMP & GCP inspections (if applicable)
Malaysia	Yes	For example, ethnicity
Zambia	Yes	Regional Information may be required for additional assessment. Some of the information*b* is assessed in the section 2.3.R Regional Information of the Drug Product Assessment Report (3.2.P) and may be subject to review

^*a*^ On the basis of the Ghana FDA reliance policy (adopted in 2019).

^*b*^ Production documentation, registration status in the country of origin, registration status in other countries, safety and efficacy bridging studies (may be required in some cases), category for distribution, summary of product characteristics, labeling (outer and inner labels), package leaflet (patient information leaflet), and advertising materials.

#### Noninnovator products other than biosimilars

Biotherapeutic products that are neither originator products nor biosimilars are approved in several developing nations.^2^ These products, referred to here as noninnovator products, can make up a substantial proportion of products on the market (see Table 6 for such terminologies and their use). In the current survey, the existence of a regulatory framework for such products in countries was assessed as well as the existence of such products on their markets. This was achieved by providing the following definition of these products: “a me‐too/non‐innovative/copy biotherapeutic product (i.e., non‐originator and nonbiosimilar) is defined as a biotherapeutic product developed on its own and not directly compared and analyzed using a licensed reference biotherapeutic product as comparator. It may or may not have been compared clinically.”^8^

**Table 6:** Terminologies and definitions used for noninnovator products that are not biosimilars

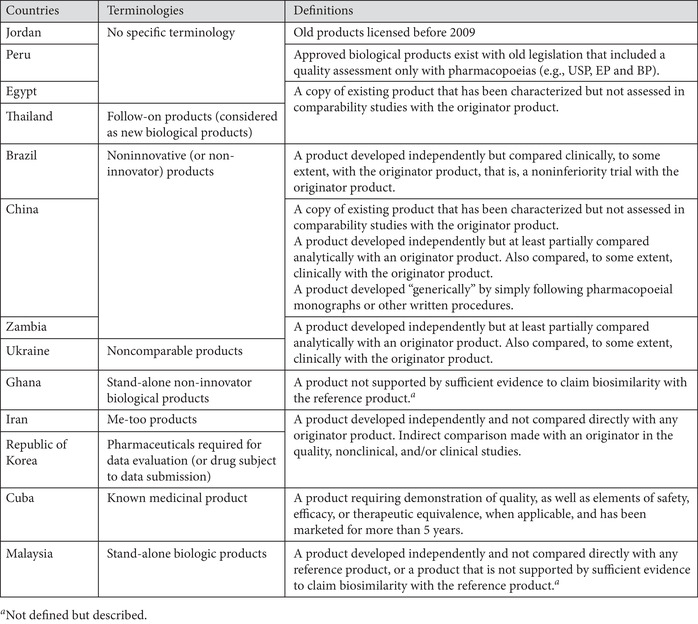

Regulations for such products have been formulated by Brazil, China, Cuba, Malaysia, Republic of Korea, and Thailand.

The situation with such products can be complex. For example, in Brazil, from 2002 until 2010, biologicals were classified as either new (innovative) or non‐new (noninnovative) products; before 2002, biological products were licensed following the same guidelines as for synthetics, adopting the same concepts and criteria. In 2010, a new regulation was published to include the concept of an SBP, but it is still possible to license noninnovative products when a comparability exercise does not apply or is not possible—this category of products is licensed through a standalone pathway.

The current (March 2020) situation with approved noninnovator products by some regulatory authorities is shown in Table 7. The dominant product class is human insulin, manufactured in various countries.

**Table 7:** Numbers of noninnovative products (but not biosimilars) approved by NRAs that have provided this information

	Brazil	China*a*	Egypt	Jordan*b*	Ukraine	Zambia
Somatropin	6	7	1	1	7	
Follicle‐stimulating hormone		2	3			
Human insulin	8*c*	14	10*d*	3	15*e*	17*f*
Insulin analog	1	4				5
Filgrastim	3	16	2	1	3	
Pegfilgrastim		2			1	
Interferon	4	23	1		15	2
Peginterferon						5
Erythropoietin	5	12	6	6	2	3
LMWH	4	11			11	6
Heparin				2		4
Infliximab					1	
Adalimumab		2				
Etanercept		3				
Rituximab		1			1	
Abciximab				1		
Bevacizumab		1				

^*a*^ Most products from China.

^*b*^ Most product registrations have been discontinued due to no submission for reregistration, with the exception of one heparin renewed in 2013.

^*c*^ 2 from Ukraine and 6 from India.

^*d*^ 10 from Egypt.

^*e*^ 7 from Ukraine, 3 from Poland, 3 from India, and 2 from Russia.

^*f*^ 10 from South Africa and 7 from India.

LMWH, low‐molecular‐weight heparin.

#### Reassessment/reevaluation of biotherapeutics already on the market

It became apparent during WHO implementation workshops^9^ that some regulatory authorities have products on their markets that were licensed some time ago using data that no longer meet current regulatory expectations or using requirements for which regulatory evaluation was not well defined. It is, therefore, possible that at least some of these products are inappropriate for current clinical use. The 14th International Conference of Drug Regulatory Authorities (ICDRA) discussed these issues and recommended the WHO to develop guidelines for evaluating these products,^10^ which the WHO has successfully achieved.^11^ This guidance outlines procedures and actions to take for dealing with products, which were approved in this manner to resolve the potential clinical issues that may arise, without leading to product shortages.

The survey showed that several countries have already taken actions to reevaluate such products on their markets. This can be summarized:

- Brazil had several such products on its market as noninnovator products (see Table 7). Among those, all that were licensed before March 2002 have been reassessed in terms of efficacy and safety for each indication, that is, four somatropins, one filgrastim, one interferon, and two erythropoietins.
- In Cuba, products are subject to renewal of their marketing authorization every 5 years, and it intends to implement the recommendations of the WHO guidance document on this from 2019.
- China has the largest number of noninnovator products on its market, some of which were approved using deficient data, but there is no plan yet for their reevaluation.
- Egypt implemented the WHO guidance document in 2015. The biological products, which were not previously assessed as biologicals, are subject to reassessment within the renewal procedure, and one insulin product has already been reassessed.
- Ghana has implemented the WHO guidance, and new national guidance for the registration of biologicals was introduced in 2013. It distinguishes requirements for biosimilars from the other versions of products, and the marketing authorization holders are required to submit the application for renewal based on the new registration guidance. When product licenses are due for renewal (every 3 years), products that do not fulfill the new requirements do not have their product registration renewed.
- Jordan has had national guidelines for biosimilar evaluation in place since 2015, and EMA guidelines were used before this (from 2009) for the evaluation of biosimilars.^2^ Biotherapeutics licensed before 2009 were re‐evaluated and new data requested from the license holder. This has resulted in the withdrawal of licenses for some products, including one somatropin, four erythropoietins, three human insulins, one filgrastim, and one abciximab, although one heparin was renewed in 2013.
- In Peru, it was evident that products approved prior to 2016 were assessed using regulatory requirements for pharmaceutical products in the absence of the regulation for biological products, as biologicals were treated the same as chemicals.^2^ In 2016, the WHO guidance on such products was adopted.
- The Republic of Korea has had an ongoing program of reevaluation of pharmaceuticals on its market since 2008.
- Singapore plans to implement the relevant WHO guidelines.
- Thailand has a policy that any medicines that are found unsafe may undergo a re‐evaluation process. In 2013, the regulatory authority decided to reevaluate all registered erythropoietin products because of the evident pure red cell aplasia (PRCA) problem.^12^ After the reevaluation, the registration of some erythropoietin products was cancelled due to the license holders providing insufficient data/documentation than that requested by the Ministerial Ordinance in 2013.
- The Ukraine regulatory authority acknowledged the need for reassessment of some products in 2018 and plans to implement the WHO guidelines.
- Zambia has published guidelines for renewal and recently initiated the renewal process.

### Issues associated with the use of biosimilars

Some important issues (e.g., intellectual property, interchangeability and substitution, and labeling) that are associated with the use of SBPs are not elaborated in the WHO Guidelines.^1^ However, questions reviewing the situation in participating countries on interchangeability and naming of biosimilars were included in the survey as they clearly affect biosimilar regulation and use.

#### Interchangeability of biosimilars

Interchangeability of biosimilars is an important, although controversial, aspect of their clinical use. There are some differences in the definitions and approaches to interchangeability in the countries and regions. Interchangeability is defined in the EU as the medical practice of replacing one medicine for another that is expected to achieve the same clinical effect; replacement of one product by another can be achieved by switching that is decided by a physician or (automatic) substitution at the pharmacy level. However, in Canada and the United States, interchangeability refers to automatic substitution but not switching. In the United States, the term *interchangeable* (or *interchangeability*) means that the biosimilar “may be substituted for the reference product without the intervention of the health care provider who prescribed the reference product.”^13^ In most of the survey‐participating countries, interchangeability refers to switching, and automatic substitution is not accepted. The complexity in details of how it is applied varies considerably depending on national frameworks for the use of biotherapeutics, including biosimilars. With the exception of the United States, where the NRA provides guidance for interchangeability, regulators in other countries are not directly involved in the decision on this. The WHO Guidelines^1^ provide science‐based guiding principles for evaluation of biosimilars with the clear statement that interchangeability should be defined at the national level by taking into consideration all relevant factors for the population in question.

The survey revealed that most countries do not have regulatory guidelines for the interchangeability of biosimilars, but many have adopted national approaches for this. In Europe, regulatory assessment by the EMA does not include any recommendation on whether a biosimilar is interchangeable with the reference product. This is because the responsibility for interchangeability is delegated to and substitution policies are within the remit of the individual EU member states. Consequently, several EU countries have issued national guidance, for example, Denmark, Finland, and the Netherlands. In addition, medical health societies in the EU have provided guidance on switching from an originator product to the corresponding biosimilar. For example, in the UK, there are no regulations/guidelines established by the regulatory authority but there are National Health Service guidelines that state: “Biosimilar products are considered to be interchangeable with their reference product; which means a prescriber can choose the biosimilar medicine over the reference product (or vice versa) and expect to achieve the same clinical effect (therapeutic equivalence).” The interchangeability is subject to the consultation between the prescriber and the patient within the national framework.^14^ In addition, guidance from the British Society of Gastroenterology states that “There is sufficient evidence to recommend that patients who are in a stable clinical response or remission on Remicade therapy can be switched to Remsima or Inflectra at the same dose and dose interval. This should be done after discussion with individual patients, with explanation of the reason for switching.”^15^ In Canada, the authorization of a biosimilar is not a declaration of equivalence to the reference biologic product. Decisions on interchangeability are made by each province and territory according to its own rules and regulations.

The current situation with interchangeability, as reported in the survey, is summarized in Table 8. Most of the countries rely on the decision made by prescribers. However, Brazil, Cuba, Ghana, Peru, Russia, and Zambia also consider the clinical evidence provided by the biosimilar manufacturers. In Brazil and Ghana, if the evidence is provided by the biosimilar manufacturer, it might be available to the prescribers as a part of the summary of product characteristics (SmPC) or the product label. In Russia, if the manufacturer of the biosimilar provides appropriate clinical evidence, the biosimilar product may be considered interchangeable. The Russian national review committee evaluates the submitted data and provides a positive decision if the relevant data/evidence are acceptable (i.e., proves that the biosimilar product can be used interchangeably). On the basis of the expert committee's decision, the regulatory authority can change the status of the biosimilar concerning its interchangeability. This serves as a recommendation for the physician to use the product interchangeably. Iran and Japan accept interchangeability automatically upon approval of the SBP.

**Table 8:** Approach for interchangeability of biosimilars in each country participating in the survey

Countries	Automatically upon approval of the biosimilar	Depending on the clinical evidence provided by the biosimilar manufacturer	Relying on the decision made by prescribers
Canada			Yes
China			Yes
Egypt			Yes
EU			Yes
India			Yes
Indonesia			Yes
Jordan			Yes
Malaysia			Yes
Republic of Korea			Yes
Singapore			Yes
Thailand			Yes
Brazil		Yes	Yes
Ghana		Yes	Yes
Zambia		Yes	Yes
Cuba		Yes	
Peru		Yes	
Russia		Yes	
Iran	Yes		
Japan	Yes		

Russia and Ukraine have regulations/guidelines on interchangeability. In Russia, the decree of the Government of the Russian Federation on the procedure for determining the interchangeability of drugs for medical use (2015) requires additional clinical study data to prove the interchangeability between a biosimilar and the RBP. In Ukraine, interchangeability is not acceptable.

#### Naming and labeling of biosimilars

Naming and labeling are clearly both very important for all biologicals, including biosimilars. These are essential for the identification of products, but also for pharmacovigilance and prescribing.

Data from the survey showed that most of the countries do not have specific regulations/guidelines relating to the naming and labeling of biologicals/biosimilars. Exceptions to this are: Canada, which established policy on this in 2019 (“Notice to Stakeholders‐Policy Statement on the Naming of Biologic Drugs”); Iran, which adopted a regulation for naming of pharmaceutical products in 2013; and Japan, which finalized nomenclature rules in 2013. The naming criterion for biologics in China is fully implemented (Supplementary volume I of The Pharmacopoeia of the People's Republic of China 2015 Edition). The Chinese regulatory authority has prepared draft guidance on naming biosimilars in 2018, but this has not yet been officially adopted.

The survey identified the following approaches being used for the naming of biosimilars:

- Many countries use the brand name and international nonproprietary name (INN) (i.e., same INN as RBP) without any other distinguisher.
- The EU refers to biosimilars by brand name or INN + marketing authorization holder, if no brand name is adopted by the marketing authorization holder.
- Japan, Malaysia, Peru, and Thailand have a distinguisher (identifier), that is, product‐specific suffix, as part of the name. In Japan, this is the INN followed by “BS” (for biosimilar) and a number; biosimilar products with the same INN are numbered sequentially as they are approved, for example, bevacizumab BS1. Malaysia has the statement “biosimilar product” on the label and prescribing information, and Peru includes the statement “similar biological product” in the summary of product characteristics. Thailand uses the letter code “NBS” followed by sequential numbering.

## Discussion: challenges and opportunities

### Reference products

Despite progress with many aspects of the development of biologicals and especially biosimilars, the survey highlighted a number of main obstacles (challenges) in developing/regulating biosimilars in each country. Many of these related to RBP issues. These include limited access to information on the RBP (comment from 13 participating countries), financial constraint due to the high price of the RBP (8 countries), and obtaining sufficient quantities of the RBP to conduct the comparability assessment (7 countries). Other problems noted were conducting bridging studies when the RBP is sourced from outside the country (six countries).

Possible solutions to these challenges include exchange of information on products between NRAs, accepting foreign‐sourced RBPs, and avoiding conducting unnecessary (duplicate) studies. Some countries have established a memorandum of understanding (MoU) to facilitate information sharing among regulatory authorities regarding the RBPs.

When the manufacturer plans the global development of an SBP and/or wants to use an RBP sourced from less‐expensive markets, unnecessary repetition of nonclinical and clinical studies already undertaken with an RBP licensed and sourced in another country needs to be avoided. For example, a version of an RBP licensed and used for biosimilarity assessment in the EU could have already had its comparability demonstrated with a version of it licensed and used in the United States.

Some regulatory authorities require bridging studies to support the use of a foreign‐sourced RBP. They must establish an acceptable bridge and a scientific basis to consider comparing the local‐ to the foreign‐sourced RBPs and include analytical comparison and sometimes clinical pharmacokinetic/pharmacodynamic studies. Bridging studies can lead to significant increase of the cost of regulatory approval. For example, biosimilar manufacturers estimate that 50–100 subjects would be expected for each comparative clinical bridging study for a biosimilar product in a jurisdiction, with the cost estimated between USD 5 and 10 million.^16^ If existing data and information are sufficient to establish the necessary bridge, the study might be unnecessary. This shift would reduce the costs of biosimilar development by waiving unnecessary or redundant studies. Even if bridging studies are limited to an analytical comparison, it would still be less expensive compared with conducting clinical trials and will contribute to lowering the barrier to market entry for a biosimilar manufacturer.

### Lack of resources

Insufficient resources of NRAs are a common problem and this is likely to continue. Lack of resources in some countries means that implementation of the WHO Guidelines is likely to be slow or may not even occur. Lack of expertise requires capacity building, which is a lengthy process. This challenge may be reduced by relying on information available from other regulatory authorities that have assessed particular products and also by joint review of applications. Work sharing and information sharing have been recognized as possible avenues for the development of expertise, particularly within the African region, as a short‐term measure.^3^ This clearly requires that regulatory authorities have confidence in each other's expertise. As the survey results show, several countries have adopted strategies for regulatory collaboration, including reliance and joint review (Table 4). Since there is evidence that the concept of reliance based on information and approval from an experienced regulatory agency in the area of evaluation of postapproval changes of biotherapeutics operates well and improves efficiency in some countries (e.g., Brazil (pilot), Ghana, and Singapore), it could offer an opportunity for an efficient and effective regulatory process for biosimilar evaluation in countries with limited resources.^17^

Regulatory authorities can facilitate access to biosimilars by reducing the time and improving the efficiency of their review without compromising its quality. Regulatory authorities, especially in countries with limited regulatory resources, should organize their activities to avoid repeating assessments/evaluations for products that have already undergone rigorous evaluation in other countries, as was recommended by ICDRA in 2014.^18^ However, efforts to build their own capacity should be undertaken as a long‐term measure. In addition, the concept of mutual or one‐way recognition in the context of regulatory evaluation for the purpose of licensing SBPs has been promoted and used in some countries. This initiative is aiming for more appropriate use of existing resources to facilitate access to SBPs of assured quality, safety, and efficacy, based on the reliance on competent authorities in the world.

WHO prequalification is a process based on the assessment of compliance to WHO standards that has been established to support countries to increase access to quality assured products.^18^ Since the WHO Guidelines are considered as a WHO‐written standard and used as a basis for the WHO prequalification, the project should be a guide for low‐ and middle‐income countries that lack regulatory resources for the assessment of biosimilars and the procurement decision. This would also explore more possibility for regulatory convergence at the global level^4^ and contribute to increasing access to quality‐assured biotherapeutic products, including biosimilars, as highlighted in the World Health Assembly resolution made in 2014.^19^ The WHO has launched a pilot project to prequalify selected biotherapeutic products, including biosimilars. This is to assist member states with insufficient capacity for assessing the quality, safety, and efficacy of biosimilars. The pilot program started with two biotherapeutic products used for cancer treatment in the WHO Essential List in July 2018 and this was expanded to include insulin in November 2019. The WHO prequalified its first biosimilar, trastuzumab, in December 2019^20^ and rituximab in May 2020.^21^

### Quality of biosimilars

A significant problem with the quality of some biosimilars (and even more with noninnovator products) was noted during the previous survey and this still continues. Some countries have products called biosimilars but that were approved prior to the establishment of a regulatory framework for biosimilars approval.^2^ WHO has recommended avoiding use of the term SBP or biosimilar for products that have not been evaluated in line with the principles in the Guidelines for these products.^1^, ^4^, ^11^ Following the advice stipulated in the WHO guideline on such problems/products^11^ should help to alleviate this problem, but this will clearly take time and needs resources. The problem of being able to clearly distinguish between real biosimilars and nonbiosimilar, noninnovator products also continues and is compounded by products being called biosimilars when they are not. The terminology used for noninnovator products should not be confused by calling them biosimilars, and such products may need to be reassessed by regulatory authorities to establish their suitability for clinical use/marketing.^2^, ^4^, ^11^ The need for an efficient and comprehensive pharmacovigilance system is crucial for postmarket surveillance. Only with such a system in place can efficacy and safety of biologicals, including biosimilars, be assured. Some important clinical issues can only become evident during such pharmacovigilance phases due to the low incidence of their occurrence. An example of this is the unwanted immunogenicity issues with erythropoietin products that leads to PRCA.^12^, ^22^ Although erythropoietin‐induced PRCA is a very serious problem, its incidence is relatively low, making it normally undetectable during clinical trials. It can, however, be detected during the pharmacovigilance stages.

### Use of biosimilars: interchangeability, naming, and pharmacovigilance

The hottest topic in biosimilars has moved on from establishing a suitable regulatory framework to the practice of interchangeability. Each regulatory authority has its own approach on the designation of interchangeability (see Table 8). In addition, the U.S. Food and Drug Administration (FDA) has adopted a unique regulatory process whereby an appropriately qualified biosimilar can be approved as interchangeable; however, requirements for this are very challenging and no such products have been approved by the FDA to date. No other regulatory authority has adopted this regulatory approach. Despite much discussion and consideration, interchangeability remains a difficult issue with biosimilars, particularly for healthcare professionals. It requires more concrete information on its efficacy and especially safety if valid conclusions on its adoption are to be made. It is, however, more of an issue to be considered and decided by physicians and patients rather than a regulatory issue. The decision to switch from an originator product to a biosimilar is based on several factors but is largely an economic issue.

Physicians and patients may be challenged by the arrival on their markets of biosimilars for which the scientific concept underlying their development and licensing is not always well understood. The role of regulatory authorities in this is to inform physicians and patients about the regulatory assessment and decision as a prerequisite for switching products on the market. Regulators have made considerable efforts to communicate with and educate all concerned, including physicians and patients, about biosimilars and their approval and advantages. Publication of a national Q&A document on biosimilars similar to that published by the WHO^7^ and public assessment reports on biosimilars could serve as potential tools for aiding such communication. As an example, a template for an assessment report titled “Public Assessment Summary Information for Biosimilars,” published by the Biosimilar Working Group of the International Pharmaceutical Regulators Forum, could be used by regulators worldwide.^23^ This communication tool would contribute to enhanced transparency and increase the public's confidence to uptake biosimilars and promote their interchangeability. As discussed above, only with an efficient and comprehensive pharmacovigilance system in place can efficacy and safety of biosimilars be assured. Thus, good pharmacovigilance is also essential for establishing the safety and efficacy of interchangeability of biosimilars.

From the perspective of the WHO, there is no specific nomenclature for biosimilars, that is, there is no part of an INN which indicates that a product is a biosimilar. Biosimilars are given INNs using the process and rules used for all biologicals, which is logical. In many cases, the INN for a biosimilar is the same as that for the reference product, for example, for GCSF biosimilars that have used Neupogen as a reference product, both the biosimilar and the RBP have the INN “filgrastim.” There has been much discussion on naming and labeling biosimilars, but to date, there is no consensus. This could potentially lead to problems with identifying products and pharmacovigilance unless careful attention is paid to the issue. This situation has caused concerns, for example, prescription mix‐ups, unintentional switching, and questions on traceability. To avoid such problems, biosimilars “should be clearly identifiable by a unique brand name” together with the INN, as stated in the Guidelines.^1^ Provision of the lot number, which is an important part of production information, is also essential.

## Conclusions

The 2010 survey identified numerous issues and problems with biosimilars and other biological products, as briefly mentioned above in the Introduction. In particular, the diversity of regulatory frameworks for licensing biosimilars and the ambiguous use of the terms *similar* or *generic* have presented significant challenges, among other issues.^6^ The 2020 survey shows that some progress with resolving all of these has been made but they all still remain issues of concern at least in some countries. The WHO Guidelines have contributed to increasing regulatory convergence at the global level, and the terminology used for biosimilars is more consistent than in the past; the term *biogeneric* seems to have been largely abandoned.^2^ However, the main challenges identified from the 2020 survey are the unresolved issues mentioned above and in the following.

Many of these challenges related to RBP issues, in particular, unavailable/insufficient RBPs in the country. Possible solutions to these challenges include: (1) exchange of information on products between NRAs to accept foreign‐licensed and ‐sourced RBPs; and (2) avoiding conducting unnecessary (duplicate) studies. Accepting foreign‐licensed and ‐sourced RBPs might contribute to expanding the availability of various product classes since these products were not available on the market prior to approval of the SBP. Some regulatory authorities require bridging studies to support the use of a foreign‐sourced RBP, but the data requirement and the rationale for requirements are not well defined. If data and information are available in the public domain and are sufficient to establish this bridge, the additional study might be waived and would result in reducing the cost of biosimilar development.

Lack of resources of NRAs is a problem and requires capacity building, which is a lengthy process. This challenge may be reduced by relying on information available from other regulatory authorities that have assessed particular products and also by joint review of applications.

All survey‐participating countries now have biosimilar guidelines in place and biosimilars approved.^2^ However, biotherapeutic products that are neither originator products nor biosimilars are also approved in several developing nations, that is, noninnovator products. A significant problem with the quality of some biosimilars (and even more with noninnovator products) was noted during the previous survey and this still continues. The terminology used for noninnovator products should not be confused by calling them biosimilars, and such products may need to be reassessed by regulatory authorities to establish their suitability for clinical use/marketing.

The practice of interchangeability and the naming of biosimilars, which are related to the use of biosimilars, are evident issues. Each NRA has its own approach to the designation of interchangeability, and there is still no consensus among countries on the naming and labeling of biosimilars, although the use of trade names for particular biosimilar products seems prevalent. Nevertheless, it is clear that naming and labeling are both very important for the identification of products and also for pharmacovigilance and prescribing. Good pharmacovigilance is essential for establishing the safety and efficacy of interchangeability of biosimilars.

Addressing and resolving many of these issues will require sharing experience and knowledge relating to biosimilars and other biologicals between NRAs. Although biosimilars have been on the market in some countries for some time and NRAs have developed considerable experience with them, this is not the case in other countries where the NRAs are relatively new to the field and clearly require more assistance and advice on all matters relating to the regulation of biosimilars.

For all regulators, evaluation of applications for biosimilar approval is challenging and product assessment difficult. Training is also problematic for those with little background knowledge and limited experience with biosimilars and particular product classes (e.g., mAbs).

For manufacturers of biosimilars, life‐cycle management is a critical issue in the context of access to biosimilars of assured quality.^4^ WHO Guidelines on postapproval changes to biotherapeutic products as well as supporting materials and technical assistance to countries have been provided.^17^, ^24^ This area requires further activity to improve the situation at the global level.

Increasing numbers of biosimilars are now available for use in clinical practice, and physicians, other prescribers, and patients are all challenged to become familiar with the advantages of the clinical use of biosimilars. They need experience with the use of them and to be made aware of real‐world information on their advantages and potential disadvantages. Information sharing and well‐documented history provided by healthcare professionals of their experience on the interchangeability of biosimilars and their use in replacement of their reference products might contribute to an increasing rate of acceptance of biosimilars for use by other professionals and patients.

## Disclosure

The authors alone are responsible for the views expressed in this article and they do not necessarily represent the views, decisions, or policies of the institutions with which they are affiliated.

## Author contributions

H.‐N.K. and R.T. assessed the answers from the survey and led the writing of the manuscript with I.K. The remaining authors are the survey participants from 19 countries who provided the answers on the survey questionnaire and contributed to validating the manuscript. The survey participants are listed in alphabetical order in the author section after the three primary authors.

## Competing interests

The authors declare no competing interests.
